# Potential Anti-Metastatic Role of the Novel miR-CT3 in Tumor Angiogenesis and Osteosarcoma Invasion

**DOI:** 10.3390/ijms23020705

**Published:** 2022-01-09

**Authors:** Lavinia Raimondi, Alessia Gallo, Nicola Cuscino, Angela De Luca, Viviana Costa, Valeria Carina, Daniele Bellavia, Matteo Bulati, Riccardo Alessandro, Milena Fini, Pier Giulio Conaldi, Gianluca Giavaresi

**Affiliations:** 1 IRCCS Istituto Ortopedico Rizzoli, CS Surgical Sciences and Technologies–SS Omics Science Platform for Personalized Orthopedics, 40136 Bologna, Italy; angela.deluca@ior.it (A.D.L.); viviana.costa@ior.it (V.C.); valeria.carina@ior.it (V.C.); daniele.bellavia@ior.it (D.B.); milena.fini@ior.it (M.F.); gianluca.giavaresi@ior.it (G.G.); 2 IRCCS-ISMETT (Istituto Mediterraneo per i Trapianti e Terapie ad alta Specializzazione), Department of Research, 90127 Palermo, Italy; agallo@ismett.edu (A.G.); ncuscino@ismett.edu (N.C.); mbulati@ismett.edu (M.B.); pgconaldi@ismett.edu (P.G.C.); 3 Department of Biomedicine, Neuroscience and Advanced Diagnostics (B.N.D), Section of Biology and Genetics, University of Palermo, 90133 Palermo, Italy; riccardo.alessandro@unipa.it; 4 Institute for Biomedical Research and Innovation (IRIB), National Research Council (CNR), 90146 Palermo, Italy

**Keywords:** osteosarcoma, microRNAs, tumor angiogenesis, metastasis, EMT proteins

## Abstract

Osteosarcoma (OS) is the most common primary bone tumor mainly occurring in young adults and derived from primitive bone-forming mesenchyme. OS develops in an intricate tumor microenvironment (TME) where cellular function regulated by microRNAs (miRNAs) may affect communication between OS cells and the surrounding TME. Therefore, miRNAs are considered potential therapeutic targets in cancer and one of the goals of research is to accurately define a specific signature of a miRNAs, which could reflect the phenotype of a particular tumor, such as OS. Through NGS approach, we previously found a specific molecular profile of miRNAs in OS and discovered 8 novel miRNAs. Among these, we deepen our knowledge on the fifth candidate renamed now miR-CT3. MiR-CT3 expression was low in OS cells when compared with human primary osteoblasts and healthy bone. Through TargetScan, VEGF-A was predicted as a potential biological target of miR-CT3 and luciferase assay confirmed it. We showed that enforced expression of miR-CT3 in two OS cell lines, SAOS-2 and MG-63, reduced expression of VEGF-A mRNA and protein, inhibiting tumor angiogenesis. Enforced expression of miR-CT3 also reduced OS cell migration and invasion as confirmed by soft agar colony formation assay. Interestingly, we found that miR-CT3 behaves inducing the activation of p38 MAP kinase pathway and modulating the epithelial-mesenchymal transition (EMT) proteins, in particular reducing Vimentin expression. Overall, our study highlights the novel role of miR-CT3 in regulating tumor angiogenesis and progression in OS cells, linking also to the modulation of EMT proteins.

## 1. Introduction

Osteosarcoma (OS) is a primary malignant tumor of bone tissue, whose incidence shows a bimodal age distribution, affecting children and adolescents (with an initial peak at 12–14 years of age) and adults (over 60th) [1]. The five-year survival rate for patients diagnosed with non-metastatic OS has increased to 70–80% through wide resection surgery combined with multi-drug adjuvant chemotherapy [2]. Nevertheless, more than 60% patients developed metastasis during treatment with 39% presenting lung metastasis [3]. Notably, the 5-year survival rate of patients who develop chemo-resistance decreased to less than 20%, supporting in turn a rapid growth of metastatic lesions [4]. The improvement in OS treatment (surgery and chemotherapy) and the advances in understanding the molecular mechanisms underlying OS pathogenesis have altogether increased the long-term survival rate. Moreover, through the identification of specific mediators of OS progression and tumor pathways, novel approaches for targeting OS are being developed. However, the bulk of our understanding of OS is based to date on anatomical and histological principles and clinical decisions are closely related to the latter [5,6,7,8]. A further issue of great importance regards micro-metastasis, which is estimated to be present in over half a percentage of patients without lung metastasis at initial examination [9,10]. Therefore, despite recent advances, discovery of novel biomarkers for OS diagnosis, prediction of response to therapy and disease progression is still necessary [11].

The discovery of microRNAs (miRNAs) would seem to represent a valid tool for an earlier diagnosis and prognosis prediction, as well as potential therapeutic targets in several cancers, including OS [12]. MiRNAs are a class of 21–25 nucleotide non-coding RNAs involved in post-transcriptional regulation of gene expression and playing pivotal role in carcinogenesis, where they contribute to control tumor proliferation [13], migration, invasion [14], epithelial mesenchymal transition [15] and angiogenesis [10,16,17,18]. Crucial factors in the control of gene regulation by miRNAs include the functionalized compartmentalization and shuttling of miRISC (the minimal miRNA-induced silencing complex) inside the cells. Notably, it has been reported that the availability and abundancy of miRNAs, such as their target mRNAs, can specifically target which gene needs to be regulated. This last consideration may explain why the suppression of mRNA targets by miRNAs is not always the same between cell types [19].

MiRNAs are not only detectable inside the tumor cells, but also in body fluids such as plasma and serum, where they can circulate free or encapsulated in microvesicles released by cancer cells [20,21,22]. Tumor circulating miRNAs are of great interest for their diagnostic and prognostic potential through easy-to-perform analysis, the so-called liquid biopsy [23]; moreover, changes in miRNAs levels seem to correlate with disease insurgence and progression. Nevertheless, several limitations are counted in miRNA studies from biofluid samples, as current methodologies need to be standardized. [24]. For all these peculiar characteristics of miRNAs, several studies mentioned in literature indicated miRNAs as potential therapeutic agents and biological biomarkers [25]. Since the interplay between miRNAs and its targeted genes is intricate and not always easy to explain, it would be appropriate to find a microRNA expression profiles, which identify, as much as possible, the disease and permit to follow its progression [23,26].

We previously investigated the miRNAs cargo from OS cells and their microvesicles by performing small RNA sequencing through NGS Illumina platform. Hierarchical clustering revealed a specific molecular profile of miRNAs and among these miRNAs, some were already known for their involvement in the tumor microenvironment establishment, bone metabolism and tumor angiogenesis [27]. Taking advantage of the NGS approach, we then discovered and analyzed the expression of 8 novel miRNAs both in OS cancer cells and a panel of human tissues. Bioinformatic analysis revealed that potentially these 8 novel miRNAs seemed to be associated with the pathogenesis and progression of human malignancies [28] and further studies will be carried out in order to understand their potential use as therapeutic targets or molecular biomarkers.

In the current study we focused our attention on the fifth candidate, renamed now miR-CT3, (pre-miR sequence chr8:129022790..129022852, mature sequence UGCGCAGUGGCAGUAUCGUAGCC), predicted to potentially regulate VEGF-A signaling pathway and other ones related to carcinogenesis [29,30]. Notably, VEGF-A is considered a poor prognostic marker for tumor-free survival in OS patients, suggesting its potential role for anti-VEGF therapy [31]. Recently, bioinformatics analysis reconstructed the interaction network of all OS-associated miRNAs and their targets and interestingly, VEGF-A resulted a part of hub gene nodes in the subnetwork of OS. In addition to the VEGF-A factor, the study highlighted the involvement of several known metastasis-related microRNAs [23]. Here we provided evidence that miR-CT3 played significant roles in migration/invasion mechanisms and regulated epithelial-mesenchymal transition (EMT)-correlated proteins. Hence, our hypothesis is that miR-CT3 could play a potential anti-metastatic role in regulating tumor angiogenesis and OS invasion.

## 2. Results

### 2.1. Novel miR-CT3 and Target Gene Prediction

**Figure 1:**
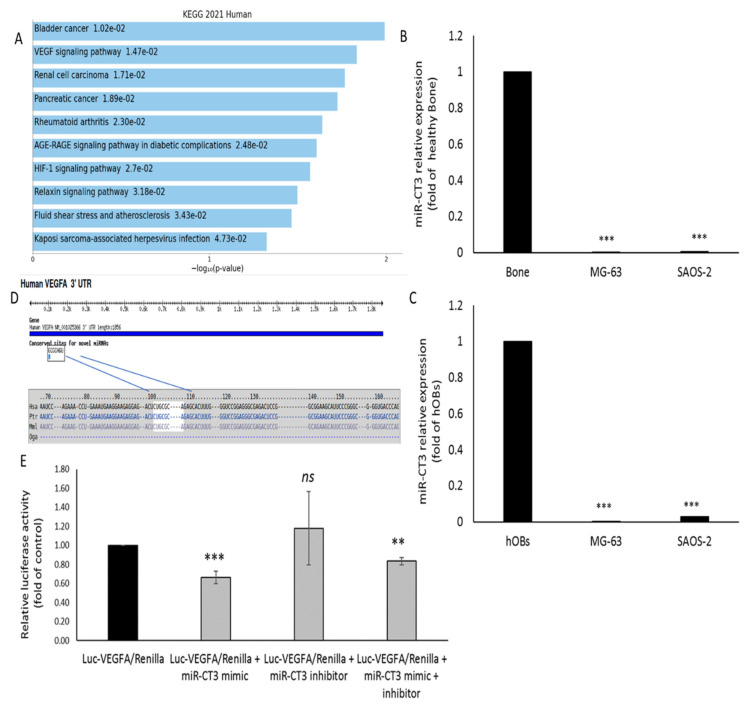
MiR-CT3 expression in OS cells and in silico analysis. (**A**) KEGG biological processes significantly enriched (pathways with *p* < 0.05) determined by Enrichr web platform (**B**) miR-CT3 expression in OS cell lines (SAOS-2 and MG-63) and human healthy bone. (**C**) miR-CT3 expression in SAOS-2, MG-63 and human primary Osteoblasts (hOBs). (**D**) Analysis output showing predicted conserved sites for miR-CT3 broadly conserved among vertebrates in 3′UTR VEGF-A mRNA determined by TargetScan7. (**E**) Dual luciferase assay of Hela cells co-transfected with firefly luciferase constructs containing the 3′UTR of VEGF-A and miR-CT3 mimic, miR-CT3 inhibitor or scrambled oligonucleotides (NC) as indicated. The firefly luciferase activity was normalized to renilla luciferase activity. The data are reported as relative luciferase activity of miR-CT-3-transfected cells as compared to the control (NC) of a total of 6 experiments from 3 independent transfections (Mean ± SD; ** *p* < 0.005; *** *p* < 0.0005).

As reported in our previously published study, we identified eight novel microRNAs, through NGS, and among these we focused our attention on the candidate 5, renamed now miR-CT3 [28]. Through Enrich web platform we determined the KEGG biologic pathways predicted to be affected by miR-CT3, thus pointing out its potential involvement in carcinogenesis (Figure 1A). MiR-CT3 expression was evaluated in two OS cell lines (SAOS-2 and MG-63), human primary osteoblasts (hOBs) and healthy bone tissue; low levels of miR-CT3 were detected in OS cells when compared with both hOBs and healthy bone tissue (Figure 1B,C). Potential binding targets for miR-CT3 and VEGF-A were predicted through bioinformatics tools by TargetScan (Figure 1D). To validate the direct interaction among miR-CT3 and the VEGF-A mRNA we performed a reporter gene assay by using a miR-CT3 mirVana mimic custom, while to perform “loss-of-function” studies in which endogenous miR-CT3 function was inhibited we used a miR-CT3 mirVana inhibitor custom. Hela cells were co-transfected with synthetic miR-CT3 mimic, inhibitor or scrambled oligonucleotides (NC), together with an expression vector carrying the 3′UTR of VEGF-A mRNA cloned downstream to the luciferase reporter gene. Figure 1E shows a lower and significant luciferase activity in cells transfected with miR-CT3 mimic as compared to the control; instead, miR-CT3 inhibitor did not exert effects on luciferase activity.

### 2.2. The Enforced Expression of miR-CT3 Down-Regulates VEGF-A

**Figure 2:**
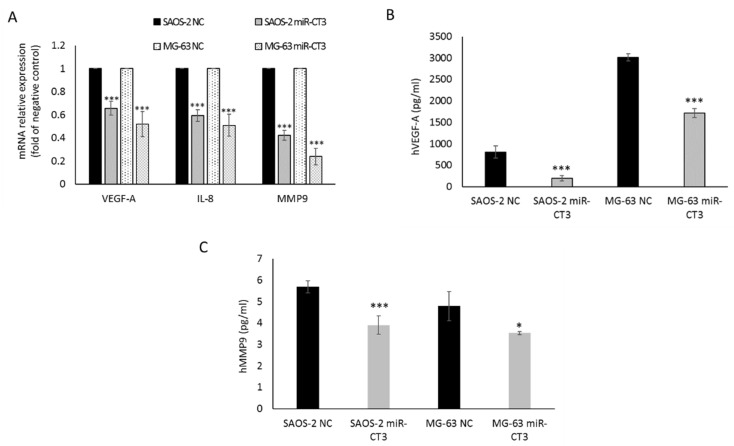
MiR-CT3 over-expression decreases expression of angiogenic and invasive markers. (**A**) Barplot of mRNA relative expression as fold of negative control (NC) of VEGF-A, IL-8 and MMP9 in SAOS-2 and MG-63 cells transfected with miR-CT3 mimics and NC. (**B**) Barplot of ELISA assay of human VEGF-A protein levels in conditioned medium of SAOS-2 and MG-63 cells transfected with miR-CT3 mimics and NC. (**C**) Barplot of ELISA assay of human MMP9 protein levels in conditioned medium of SAOS-2 and MG-63 cells transfected with miR-CT3 mimics and NC. (Mean ± SD, *n* = 3 triplicate; * *p* < 0.05; *** *p* < 0.0005).

To assess whether miR-CT3 directly affects the expression of VEGF-A in OS cells, we evaluated the effect induced by enforced expression of synthetic miR-CT3 mimics on VEGF-A protein and mRNA levels. Firstly, the transfection efficiency of miR-CT3 was measured by qRT–PCR 24 h after treatment in SAOS-2 and MG-63 cells (Supplementary Figure S1). In addition, it was observed that the enforced expression of synthetic miR-CT3 mimics reduced the expression of VEGF-A mRNA and the secretion of VEGF-A protein (Figure 2A,B). Then, the expression of the angiogenic factor IL-8 by qRT-PCR and of the metalloproteinase MMP9 by both qRT-PCR and Elisa assay were also evaluated (Figure 2A,C).

### 2.3. The Enforced Expression of miR-CT3 Impairs Vessel Formation and Adhesion of OS Cells on Vascular Endothelium

**Figure 3:**
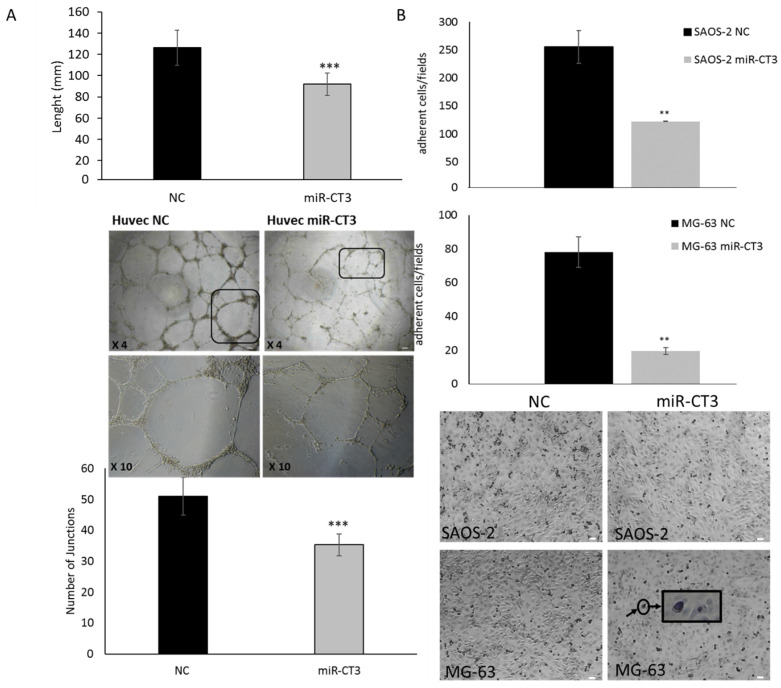
MiR-CT3 over-expression inhibits vessel formation and adhesion of OS cells on HUVEC cells. (**A**) Tube formation assay of HUVEC cells seeded on matrigel and treated for 24 h with the conditioned medium from SAOS-2 cells transfected with miR-CT3 mimics and NC. Barplots show the mean ± SD of the total length of tubes (top panel) and the number of junctions of tubes (bottom panel) per field quantified by counting 3 random fields/well under the microscope (4×) (*** *p* < 0.0005). In the middle a representative image of HUVEC cells treated as described above. (**B**) Adhesion assay of SAOS-2 and MG-63 cells transfected with miR-CT3 mimics and NC and seeded on to HUVEC cells monolayer (bottom representative image: the arrow indicates an OS cell (round and dark) adhered to the Huvec monolayer. Barplots show the mean ± SD of adherent cells per field by counting 3 random fields/well under the microscope (4×) (** *p* < 0.005).

To measure the ability of OS cells overexpressing miR-CT3 to influence endothelial cells to form capillary-like structures, tube formation assay was assessed. HUVEC cells were seeded on Matrigel^®^ (Corning, Somerville, MA, USA) and cultured in presence of conditioned medium from SAOS-2/miR-CT3 transfected cells. Tube formation by endothelial cells was visualized using a phase contrast inverted microscope and the length and junctions of tubes were quantified by using the NIS Elements Software (Nikon Europe B.V., Amsterdam, The Netherlands). As shown in Figure 3A, over-expression of miR-CT3 in OS cells perturbed tube formation and cellularity of structures, in contrast with the negative control. In particular, over-expression of miR-CT3 determined a reduction in the length (Figure 3A top graph) of the tubules and in the number of the junctions (Figure 3A bottom graph). We then verified if HUVEC cells could be affected by miR-CT3 potentially transported by exosomes contained in the conditioned medium from SAOS-2/miR-CT3 transfected cells. Supplementary Figure S4 revealed that exosomes derived from SAOS-2/miR-CT3 contained more miR-CT3 than exosomes from SAOS-2/NC (Figure S4A). Supplementary Figure S4B demontrated that exosomes from SAOS-2/miR-CT3 expressed miR-CT3.

To investigate the role of miR-CT3 in the adhesion ability of OS cells onto a HUVEC cells monolayer, we performed an adhesion assay. OS cells trasfected with miR-CT3 showed a lower capability to adhere onto endothelial cells in comparison to NC-trasfected OS cells (Figure 3B).

### 2.4. The Enforced Expression of miR-CT3 Seems Not to Regulate Cell Proliferation and Apoptosis

**Figure 4:**
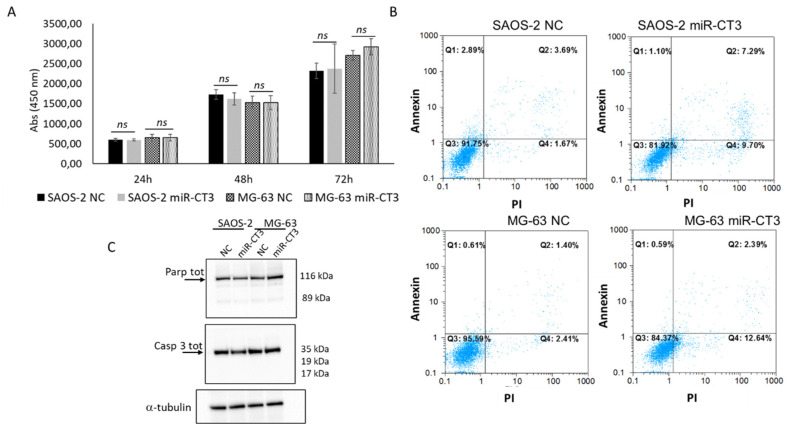
MiR-CT3 over-expression does not affect proliferation and apoptosis of OS cells (**A**) WST-1 proliferation assay of SAOS-2 and MG-63 cells transfected with miR-CT3 mimics and NC and analyzed after 24, 48 and 72 h post transfection (Mean ± SD, *n* = 3 triplicate; ns: not significant, *p* < 0.5). (**B**) Flow cytometric analysis of SAOS-2 and MG-63 cells, transfected with miR-CT3 mimics and NC, stained with Annexin V-FITC and PI. (**C**). Western blotting analysis of Parp total and Caspase3 total in SAOS-2 and MG-63 cells transfected with miR-CT3 mimics and NC. Tubulin was used as loading control.

We investigated the effects of miR-CTR3 overexpression in SAOS-2 and MG-63 cells on proliferation and apoptosis. As reported in Figure 4A, overexpression of miR-CT3 in SAOS-2 and MG-63 cells does not affect cell proliferation, from 24 to 72 h. Moreover, we also evaluated the proliferation of HUVEC cells cultured in the presence of conditioned medium from SAOS-2 and MG-63/miR-CT3 transfected cells; data confirmed those regarding OS cells (data not shown).

Double staining of the transfected OS cells with Annexin V-FITC and PI showed that miR-CT3 not significantly altered the percentage of apoptotic cells (Figure 4B). To confirm flow cytometric analysis results, western blotting analysis of the pro-apoptotic proteins Casp 9 and PARP was performed. As shown in Figure 4C (Supplementary Figure S3: densitometric analysis), miR-CT3 overexpression in SAOS-2 and MG-63 did not cause the cleavage of Casp 9 and PARP proteins, not inducing the activation of the pathway.

### 2.5. MiR-CT3 Reduces Migration and Invasive Ability of OS Cells

**Figure 5:**
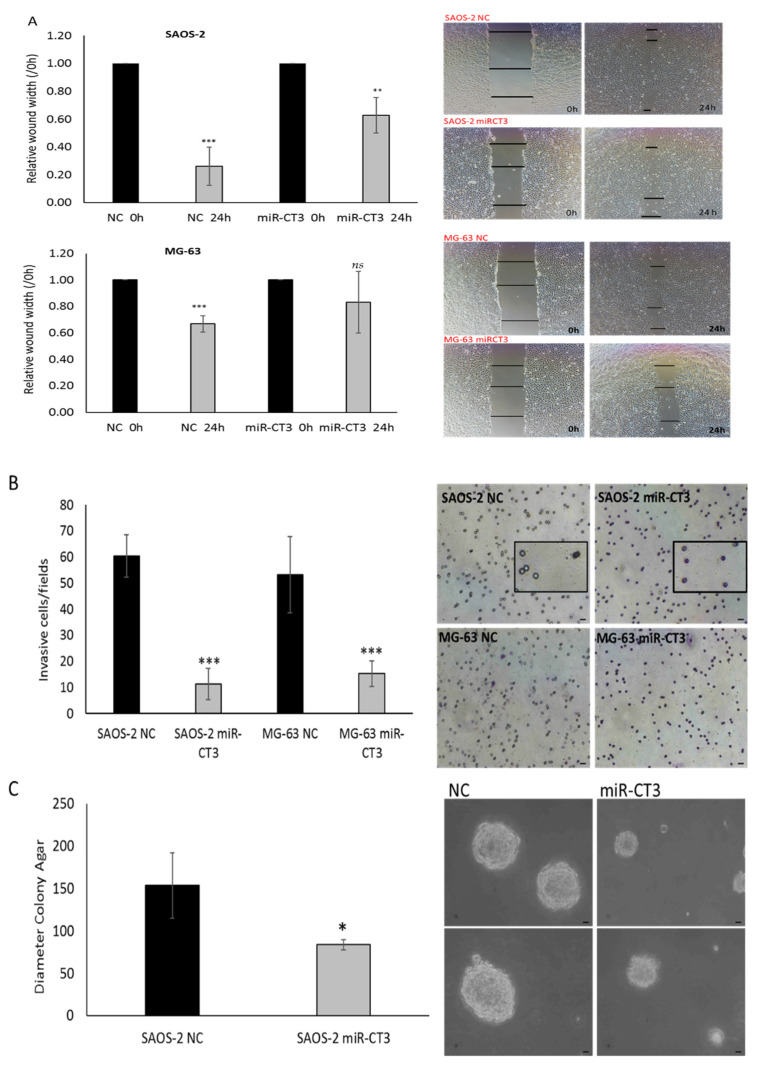
MiR-CT3 over-expression reduces invasiveness in OS cell lines. (**A**) Wound healing assays on the confluent layers of SAOS-2 (up) and MG-63 (down) cells transfected for 24 h with miR-CT3 mimics and NC. On the right of the panel: representative images of wound healing assays were acquired at 0 and 24 h after wounding, using a Nikon Eclipse Ti microscope. Data were calculated, for each condition, as the average of the widths of the gaps and normalized to initial wound width made at time oh. (**B**) Invasion assay of SAOS-2 and MG-63 cells transfected with miR-CT3 mimics and NC and seeded, after 24 h, in the upper of 24-well transwell inserts with 8 μm pore filters Matrigel-coated. Invasive cells were counted in randomly selected visual fields under the microscope. As shown in the enlarged panel (SAOS-2 miR-CT3), after 6 h the OS-miR-CT3 cells that migrate to the lower surface through the pores of the inserts appeared round and colored; after 6 h most of the OS-NC cells have already passed to the well of the plates (lower compartment) and the pores appeared empty (SAOS_NC) (**C**) Soft agar colony formation assay of SAOS-2 and MG-63 cells transfected for 24 h with miR-CT3 mimics and NC, suspended in medium containing 0.35% Noble agar and plated on a layer of 0.7% Noble agar in DMEM-10%FBS. On week 4, images (10×) were acquired and the average of the diameter of colonies was calculated (Mean ± SD, *n* = 3 triplicate; * *p* < 0.05; ** *p* < 0.005; *** *p* < 0.0005; ns: not significant, *p* < 0.5). Scale bars = 35 μM.

Wound healing assay was performed to assess the effect of enforced expression of synthetic miR-CT3 mimics on OS cells migration. SAOS-2 and MG-63 cells were scratched after 24 h of miR-CT3 transfection and evaluated for cell migration within the next 24 h. Notably, we found that miR-CT3 slowed cell migration, particularly for MG-63 cells (Figure 5A). Similarly, the number of SAOS-2 and MG-63 cells that invaded across the 8-µm pore-size membrane, Matrigel coated, decreased when cells were transfected with synthetic miR-CT3 mimics (Figure 5B). Finally, a soft agar colony formation assay was performed to evaluate the anchorage-independent growth capacity of OS cells after synthetic miR-CT3 mimic transfection to detect their tumorigenic and metastatic potential. As shown in Figure 5C, the colonies were on average with a smaller size.

### 2.6. MiR-CT3 Regulates Expression of Proteins Correlated with Migration

**Figure 6:**
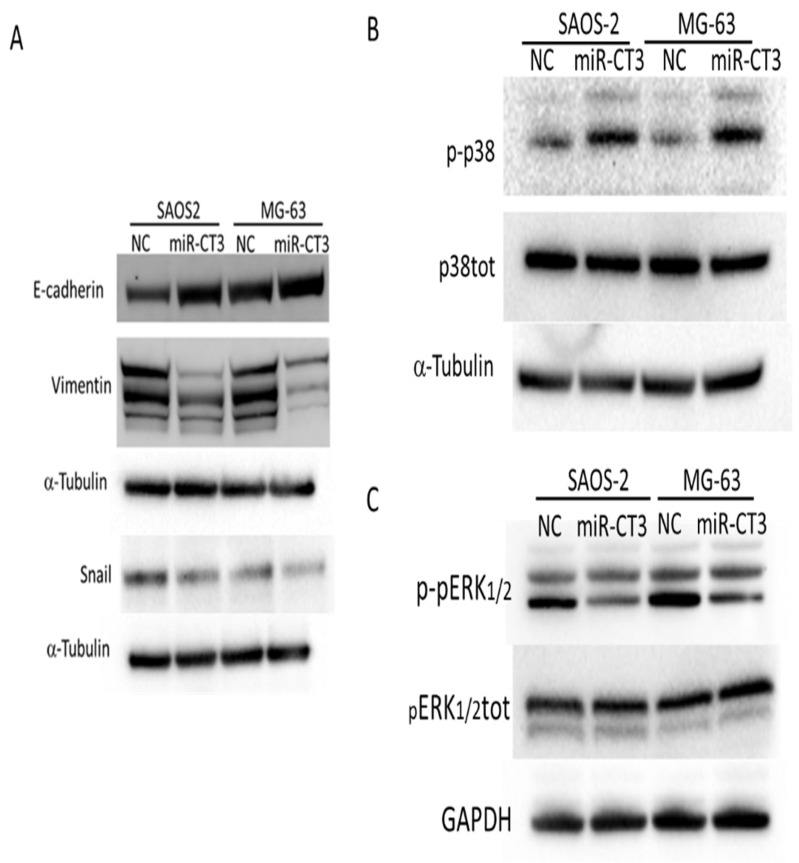
MiR-CT3 over-expression regulates the expression of proteins of the EMT-pathway. (**A**) Western blotting analysis of E-cadherin, Vimentin, Snail in SAOS-2 and MG-63 cells transfected with miR-CT3 mimics and NC for 24 h. Tubulin was used as loading control. (**B**) Western blotting analysis of phospho- and total-p38 MAPK in SAOS-2 and MG-63 cells transfected with miR-CT3 mimics and NC for 24 h. Tubulin was used as loading control. (**C**) Western blotting analysis of phospho- and total-p42/44 ERK in SAOS-2 and MG-63 cells transfected with miR-CT3 mimics and NC for 24 h. GAPDH was used as loading control.

We then investigated the potential role of miR-CT3 in regulating expression of EMT-factors. MiR-CT3 overexpression down-regulated the expression of Vimentin in both OS cell lines analyzed (Figure 6A). Accordingly, Snail expression was reduced, while E-cadherin expression increased only in SAOS-2 cell line (Figure 6A). Densitometry analysis is showed in Supplementary Figure S2.

Afterwards, we investigated whether miR-CT3 overexpression could had an effect on p38 and pERK phosphorylation levels, as it is known a correlation among the activation of these molecular pathways and invasiveness and metastasis mechanisms of cancer cells. Interestingly, miR-CT3 enforced expression in SAOS-2 and MG-63 cells induced activation of the p38MAPK signaling pathway (Figure 6B); instead, overexpression of miR-CT3 caused a reduction in phosphorylated ERK1/2 proteins (Figure 6C) Densitometric analysis is showed in Supplementary Figure S2.

### 2.7. MiR-CT3 Expression in Metastatic Patient Samples

**Figure 7:**
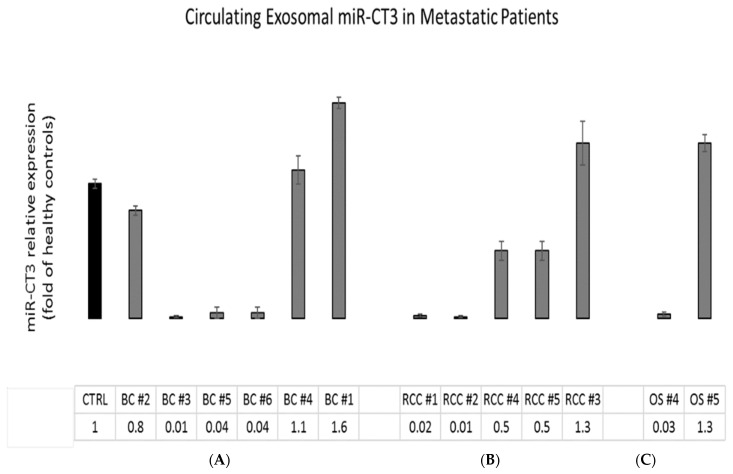
MiR-CT3 expression in metastatic cancer patient samples. (**A**) Barplots represent the circulating exosomal miR-CT3 expression in metastatic cancer patient samples (**B**,**C**)-Breast Cancer; RCC-Renal Cell carcinoma and OS-Osteosarcoma). Values are expressed as fold of healthy controls (CTRL). Mean ± SD.

Preliminary investigations on the expression of miR-CT3 in circulating microvesicles purified from collected plasma of few metastatic cancer (breast cancer and renal cell carcinoma) and OS patients, highlighted that 8 out of 13 patients presented lower miR-CT3 expression values in comparison to healthy control (Figure 7).

## 3. Discussion

Metastasis remains the principal cause of cancer death and development of therapeutic treatments for its prevention or resolution is crucial; in the current scenario of the potential therapeutic targets, miRNA-based gene therapy is certainly among the most discussed.

OS is still considered a deadly malignant tumor affecting adolescents and young adults. At diagnosis, a high percentage of OS (about ~95 percent) are classified as high-grade neoplasms, with the presence of microscopic metastatic disease, although not always detected in the right time. Conversely, intermediate, and low-grade variants are much less frequent. The main sites of metastasis are the lungs, followed by bones and lymph nodes; of note, the patient survival with lung metastasis is strongly reduced [32,33,34,35].

Aberrant expression of miRNAs has been associated with OS metastasis and the molecular mechanism of metastasis by the dysregulated miRNA needs to be fully elucidated [10]. Indeed, increasingly studies reported the role of miRNAs in regulating key steps of metastatic processes and development of a miRNAs signature for OS progression could be used in order to hamper aggressive (metastatic) biological behavior of OS [10,23].

Metastasis involves multiple steps characterized, among others, by: (1) cancer cells attracting blood vessels in the primary lesion (site) supply themselves with oxygen and nutrients; then, (2) cancer cells, interacting with the endothelium and, through circulation, they can adhere locally and, finally leaving the vessel, colonize the new tissue [10,36,37,38].

Among the miRNAs-related to invasion and metastasis of OS cells, miR-134 is indicated as a tumor suppressor miRNA in OS; by targeting VEGF-A/VEGFR1 signaling, miR-134 counteracts tumor progression and angiogenesis in OS cells [16]. Notably, an inverse correlation was reported between miR-374b and VEGF-A expression in human OS primary tumors [39]. In OS cells, aberrant over-expression of angiopoietin 2, a positive regulator of angiogenesis, was shown to be triggered by a decrease in miR-543 expression [40]. A reduction in the expression of miR-183 was correlated with lung metastases and local recurrence of OS; by in vitro assays, it was found that the tumor suppressor miR-138 worked through inhibition of Ezrin expression and suppression of MAPK/ERK activation, thus preventing cell migration and invasion of OS cells [41,42]. MiR-210-5p, instead, was found over-expressed in OS. By downregulating the expression of PIK3R5 and regulating the AKT/mTOR signaling pathway, miR-210-5p promoted the cellular process called epithelial–mesenchymal transition (EMT), which is characterized by cells progressively losing epithelial features and acquiring mesenchymal properties, peculiar aspects of invasive and metastatic cells [43]. An oncogenic miRNA, member of the miR-17-92 cluster, was upregulated in OS cells and negatively regulated SPRED2, thus promoting the malignant transformation of osteosarcoma cells [44].

We previously investigated the miRNAs cargo from OS cells and their microvesicles by RNA sequencing methods; notably, a unique molecular profile of exosomal miRNAs was found, demonstrating their involvement in osteoclast differentiation, bone resorption activity and angiogenesis [27]. In a second study, we discovered and analyzed the expression of eight novel miRNAs in OS through NGS approach [28]. In the current study, the functional role of one of these novel miRNAs, miR-CT3, was deeply investigated.

The expression level of miR-CT3 was low in OS cells (SAOS-2 and MG-63) and down-regulated when compared to human OBs and healthy bone tissue. In silico analysis revealed that the biological pathways affected by miR-CT3 were potentially related to carcinogenesis. In particular, some of these molecular pathways caught our attention, such as VEGF, HIF-1, focal adhesion and proteoglycans signaling pathways, as their aberrant regulation drives metastasis, invasion, and other processes of all malignant tumors [45].

Firstly, we focused our attention on tumor angiogenesis and through TargetScan analysis discovered VEGF-A as a potential direct target of miR-CT3; luciferase assay confirmed the predicted binding and the expression of both VEGF-A mRNA and protein resulted reduced in OS cells transfected with a miR-CT3 mimic. Secondly, we investigated the ability of OS cells overexpressing miR-CT3 to influence HUVEC cells to form capillary-like structures; notably, miR-CT3 perturbated the vessel formation, above all acting on the lengths of tubes and the number of junctions. In general, we observed the formation of capillary-like structures more subtle, weak and badly organized. Notably, we verified an increased expression of miR-CT3 in exosomes isolated from the conditioned medium of OS cells transfected with miR-CT3. Anyway, even if it seems that miR-CT3 can be transferred by exosomes, we assume that OS cells, in which we enforced miR-CT3 expression, acquire a less invasive phenotype which in turn influence the cells of tumor microenvironment through a regulated secretion of crucial factors. Then, since adhesion between tumor cells and endothelium represents a decisive step in metastasis, we looked over the capability of OS cells overexpressing miR-CT3 to adhere to the HUVEC monolayer. The enforced expression of miR-CT3 impaired the adhesion of OS cells on vascular endothelium; however, flow cytometric analysis excluded the involvement of the adhesion molecules VCAM-1(CD106) and Ep-CAM (CD326) (data not shown) and future studies will aim to investigate the key molecules involved. Notably, miR-CT3 enforced expression reduced the migration and invasion of OS cells. Subsequently, the tumorigenic potential of OS cells overexpressing miR-CT3 was evaluated through soft agar colony formation assay, a method which assesses the anchorage-independent growth ability of cancer cells. Of note, miR-CT3 overexpressing OS cells formed smaller colonies. At this point, we analyzed the expression of key molecules in metastatic processes and some molecular pathways important in cellular transformation.

Nonetheless, when the potential effect of miR-CT3 on cell proliferation and apoptotic mechanisms was analyzed, no significant changes were found. Although most miRNAs deregulated in tumors can influence different processes of malignant cells, the importance of a specific signature for miRNAs closely related to metastasis has recently been emphasized. Activation of metastatic programs implicated a specific molecular profile, which guarantees metastatic cells to acquire special properties; metastasis is driven by peculiar biochemical alterations, genetic and metabolic profiles, such an epigenetic plasticity, which can regulate metastatic development by acting on chromatin. Therefore, the same miRNA can be expressed or inhibited in a specific stage of the tumor, acting on specific molecular mechanisms, as well as having dual roles according to the cellular context and/or the tumor.

Altogether, our data indicated a marked role of miR-CT3 in controlling the metastatic potential of OS cancer, not only tumor angiogenesis, but also migratory and invasive activities were affected by miR-CT3. At this point, we analyzed the expression of key molecules in metastatic processes.

Matrix metalloproteinases (MMPs) are enzymes which degrade the extracellular matrix (ECM) and among these, MMP-9 is reported to promote the migration and infiltration of OS cells [46,47,48]. To further support the potential anti-metastatic role of miR-CT3, we observed a reduction in the MMP9 mRNA and protein expression when OS cells were transfected with miR-CT3 mimics.

EMT is involved in a wide array of malignant behaviors of cancers, including proliferation, invasion, and metastasis. During several steps of metastasis, the cancer cells disseminate into other organs via EMT. Furthermore, it is well known that miRNAs play a pivotal role in the regulation of EMT phenotype, also in OS cells [43,49,50,51]. Hence, we investigated the potential role of miR-CT3 in regulating expression of EMT-factors.

In particular, we first analyzed the expression of Vimentin, a type III intermediate filament predominantly expressed in mesenchymal cells and involved in key cellular processes such as cell motility, migration and adhesion. Vimentin drives EMT and metastasis in several solid cancers; upregulation of Vimentin is associated with poor clinical outcome in several cancers as acute myeloid leukemia [52], breast cancers [53] and oral squamous cell carcinomas [54]. Several studies have demonstrated that vimentin contributes to angiogenesis and the use of regulators of tumor angiogenesis (such as PARP inhibitors) leads to the reduction of vimentin expression and suppression of tumor angiogenesis in vascular endothelial cells [53,55]. Recently, it has been reported that cell plasticity regulated by EMT transition transcription factors (EMT-TFs) is crucial to keep the mesenchymal status and support cell invasion and metastasis of OS cells [56]. Beyond Vimentin, key factors such as Snail and E-cadherin have been described in OS cells; knockdown of Snail2 resulted in reduced motility and correlated with changes in the polymerization of the actin cytoskeleton and in focal adhesions as well as altered expression of Wnt5a, sFRP2 and osteoblast cadherin (OB-Cad) [57,58]. Furthermore, the expression of E-cadherin was described as prognostic value in OS patients [59]. Notably, in both OS cells overexpressing miR-CT3, the expression of Vimentin resulted strongly reduced; in addition, Snail expression was reduced while E-cadherin expression increased only in the SAOS-2 cell line.

Recent studies demonstrated that the deregulation of Raf/MEK/ERK signaling pathway may control tumor proliferation, migration, and metastasis in OS, and is associated with lung metastasis of OS in an orthotopic mouse model. The expression and activation of the MAPK pathway correlated with prognosis of several cancers [60,61]. Moreover, as it is reported that the MAPK/ERK pathway can be activated by the growth factor VEGF-A [60], we analyzed it in OS cells overexpressing miR-CT3 and we found that miR-CT3 significantly reduced protein levels of phospho-extracellular signal-regulated kinase (P-ERK).

The role of p38 in cancer was extensively studied. As demonstrated by several experimental data, p38 MAPK can act as a tumor promoter or tumor suppressor; these effects may depend on the stimulus received and on the cellular context. For example, it appears that p38 activation may suppress oncogenesis, and when p38 activity was forced in rhabdomyosarcoma cells, it induced terminal differentiation. Interestingly, accumulating evidence confirmed the important role of the MAPK signaling pathway in OS [62,63]. Noteworthy, it was demonstrated that silencing of long-non-coding RNA ANCR inhibited the migration and invasion of OS cells through activation of the p38MAPK signaling pathway [64].

Interestingly, we found that miR-CT3 stimulates the p38 MAPK signaling pathway in miR-CT3/OS cells.

From the evidence obtained, we hypothesize an anti-metastatic role of the novel miR-CT3, with a particular effect on invasiveness and tumor angiogenesis. We also analyzed the expression of miR-CT3 from circulating microvesicles of metastatic patients. Very preliminary data for the limited number of tumor samples showed a lower expression of miR-CT3 when compared to the healthy control, even if in some patients this was not evident. The differential expression of miRNAs detected in serum from patients may be associated with specific molecular and histological subtypes. Notably, not only patient selection and classification, is a critical issue for clinical studies; in fact, sample collection and processing as well as the variability in circulating miRNA levels in response to pharmacological treatment are important points to be seriously considered [65,66,67]. Further studies will be required to validate the potential clinical utility of this novel miRNA using larger cohorts and collecting tumor samples, distinguishing them from metastatic and not metastatic OS patients, and evaluating the expression in other tumors. In our opinion, a differential expression of miR-CT3 exists and it is related to the histological subtype and the presence or absence of metastases. Furthermore, experiments will be performed in order to better elucidate the regulation of miR-CT3 expression in cancer cells, with particular regard to the possibility of epigenetic regulatory mechanisms.

## 4. Materials and Methods

### 4.1. Cell Lines and Reagents

SAOS-2 and MG-63 cell lines were purchased from The European Collection of Authenticated Cell Cultures (Sigma–Aldrich, Italy) and grown in Dulbecco’s modified Eagle’s medium (DMEM) high glucose (Gibco, Life Technologies, USA) supplemented with 10% fetal bovine serum (FBS, Lonza Group, Basel, Switzerland). HUVEC/TERT2 hTert immortalized umbilical vein endothelial cells (HUVEC) were purchased from ATCC^®^ (LGC Standards S.r.l. Sesto San Giovanni) and cultured in vascular cell basal medium (ATCC PCS-100-030), supplemented with Vascular Endothelial Cell Growth Kit-VEGF (ATCC PCS-100-041). Hela Cells were purchased from ATCC^®^ (LGC Standards S.r.l. Sesto San Giovanni) and cultured in DMEM high glucose (Gibco, Life Technologies, USA) supplemented with 10% fetal bovine serum (FBS, Lonza Group, Basel, Switzerland). Normal human osteoblasts were purchased from Lonza (Catalog#: CC-2538) and cultured with OGMTM Osteoblast Growth Medium BulletKit™ (Catalog #: CC-3207). Conditioned medium from OS cells transfected for 48 h with synthetic miR-CT3 or scrambled oligonucleotides (NC) was obtained by centrifugating medium at 1000× *g* for 10 min. Tissue Total RNA from Bone tissue was purchased from Origene (OriGene Technologies GmbH, Rockville, MD, USA).

### 4.2. Exosome Purification

Microvesicles from plasma of metastatic patients were isolated with Total Exosome Isolation Kit (Thermo Fisher Scientific, Waltham, MA, USA), according to the manufacturer’s instructions. Microvesicles released by OS cells (SAOS-2 transfected with miR-CT3 mimic or NC) during a 24-h culture period were isolated from conditioned culture medium with Total Exosome Isolation Reagent (from cell culture media) (Thermo Fisher Scientific, Waltham, MA, USA), according to the manufacturer’s instructions.

### 4.3. Dual-Luciferase Reporter Assay

VEGFA was predicted as a potential target of miR-CT3 by using miRNA database (TargetScanHuman 7.2). The human 3′UTR of VEGFA were cloned in pMir-Targets (PS100062) vector and purchased from Origene (OriGene Technologies GmbH, Rockville, MD, USA). The reporter plasmid and miR-CT3 mimic/inhibitor or NC were co-transfected into Hela cells-293T cells using Lipofectamine 3000 (Thermo Fisher Scientific, Cambridge, MA, USA) to determine if VEGFA is a direct target. Firefly and Renilla luciferase activities were measured 48 h after transfection using the Dual-Glo R Luciferase Assay System (Promega, Madison, WI, USA).

### 4.4. Viability Assay (WST-1 Test)

Water Soluble Tetrazolium Salts (WST-l) colorimetric reagent (Roche Diagnostics GmbH, Manheim, Germany) was used to evaluate cell viability. Briefly, WST-1 reagent (10% *v*/*v*) was added to the cell monolayer in each well and incubate for 4 h at 37 °C and 5% CO_2_; the formazan dye produced by viable cells was quantified spectrophotometrically at 450 nm by Bio-Rad Microplate Reader (Bio-Rad Laboratories, Hercules, CA, USA).

### 4.5. MiRNA Transfection and qRT-PCR of mRNAs and miRNAs

Total cellular RNA was extracted using TRIzol Reagent (Invitrogen, Life Technologies, Carlsbad, CA, USA). qRT–PCR was used to confirm the expression levels of mRNAs and miRNAs. For mRNA detection, total RNA was extracted by both OS cells and HUVEC cells. For mRNA detection, oligo-dT-primed complementary DNA was obtained using the High-Capacity cDNA Reverse Transcription Kits (AB Applied Biosystems, MA, USA) and then used as template to quantify levels by Fast SYBRR Green Master Mix (AB Applied Biosystem, MA, USA). Real-time PCR was performed in duplicates for each data point. Relative changes in gene expression between control and treated samples were determined with the ΔΔCt method. Final values were expressed as fold of induction. Human primers are listed in Supplementary Table S1.

For miRNA cell transfection, Lipofectamine 3000 (Thermo Fisher Scientific, Cambridge, MA, USA) was used. In detail, OS cells were seeded at 100,000 cells/cm^2^ and transfected with 15 pmol/mL miR-CT3 mirVana miRNA mimic or inhibitor custom (Assay ID CT322JY, Life Technologies, Carlsbad, CA, USA), or scrambled negative control (4464058, mirVana negative control Life Technologies, Monza, Italy).

For miRNA detection, total RNA was extracted by OS cells after 48 h of transfection or by microvesicles isolated from plasma of metastatic patients using TRIzol Reagent (Invitrogen, Life Technologies, Carlsbad, CA, USA). Total RNA was reverse transcripted according to the manufacturer’s instructions (cat.4366596; TaqManMicroRNA Reverse Transcription, Applied Biosystems). TaqMan microRNA Assay was used to detect and quantify mature miR-CT3 (Custom Taqman Small RNA Assay; catalog# 4398987, ID#: CT322JY). MiR-CT3 expression was normalized on U6 (Applied Biosystems, Assay Id 001973 cat.4427975). Changes in the target miRNA content relative to the housekeeping U6 were determined with ΔΔCt method.

### 4.6. Western Blot Analysis

SDS-PAGE Electrophoresis and Western Blotting were performed. Briefly, cells were washed in PBS and lysed for about 30 min in lysis buffer containing 15 mM Tris/HCl pH 7.5, 120 mM NaCl, 25 mM KCl, 1 mM EDTA, 0.5% Triton ×100 and Protease Inhibitor Cocktail (100×, Sigma-Aldrich, St. Louis, MO, USA). Cell lysates (30 µg per lane) were separated using Bolt Bis-Tris gel 4–12% (Thermo Fisher Scientific, Cambridge, MA, USA) and transferred on Nitrocellulose membranes (GE Healthcare, Milan, Italy); the membrane was incubated in blocking solution (5% BSA, 20 mM Tris, 140 mM NaCl, 0.1% Tween-20) and probed overnight with the specific antibodies.

The antibodies against the following proteins were used: E-Cadherin (24E10) Rabbit mAb 3195, Vimentin (D21H3) XP^®^ Rabbit mAb 5741, Snail (C15D3) Rabbit mAb #3879, p38 MAPK Antibody #9212 and Phospho-p38 MAPK (Thr180/Tyr182) Antibody #9211, PARP Antibody 9542, and Apoptosis Antibody Sampler Kit #9915 all from Cell Signaling (Cell Signaling, Beverly, MA, USA); α Tubulin TU-02 (sc-8035) from Santa-Cruz Biotechnology (Santa Cruz Biotechnology, Inc. Dallas, TX, USA). The membranes were incubated with secondary antibody Dylight 488 (Thermo Fisher Scientific, Cambridge, MA, USA) and signal was detected by Chemidoc (Biorad, Milan, Italy).

### 4.7. Tube Formation Assay

HUVEC cells (15,000 cells/wells) were seeded on Matrigel^®^ (Corning, Somerville, MA, USA), in 96 multiwells plate and treated with supernatants of OS cells transfected with miR-CT3 mimic or NC. Tube length was measured following incubation for 24 h at 37 °C in 5% CO_2_. Images were captured using a Nikon Eclipse Ti microscope (Nikon Europe B.V., Amsterdam, The Netherlands). Three fields were photographed randomly in each well and the tube length was quantified using the NIS Elements Software (Nikon Europe B.V., Amsterdam, The Netherlands).

### 4.8. Invasion Assay

SAOS-2 and MG-63 cells transfected with miR-CT3 mimic or NC were suspended in serum-free DMEM high glucose medium supplemented with 0.1% BSA and seeded in the upper of 24-well transwell inserts with 8 μm pore filters (Corning, Somerville, MA, USA) coated with Corning Matrigel Growth Factor Reduced (GFR) Basement Membrane Matrix (Corning, Somerville, MA, USA). The lower chamber was filled with 500 μL of DMEM high glucose supplemented with 10% FBS. After incubation for 6 h, the invasion capability of osteosarcoma cells was evaluated. The upper chambers were removed, fixed and stained using the Differential Quick Staining Kit (Electron Mycroscopy Sciences, EMS, Hatfield, PA UK). Cells were counted in randomly selected visual fields of an inverted fluorescence microscope at 20× magnification.

### 4.9. Wound Healing Assay

SAOS-2 and MG-63 cells transfected with miR-CT3 mimic or NC were cultured into 6-well plates. When the cell confluence reached 90%, a 10 µL pipette tip was used to scrap across the confluent cell layer, followed by gentle washing with phosphate-buffered saline (PBS), and addition of DMEM high glucose supplemented with 10% FBS. Images were captured at 0 and 24 h using a Nikon Eclipse Ti microscope (Nikon Europe B.V., Amsterdam, The Netherlands). Wound healing ability was determined by measuring the scraped area alteration due to cell migration using Image J software [68].

### 4.10. Adhesion Assay

HUVEC cells were grown to confluence in 12-well plates and fixed with glutaraldehyde 0.0125% (Agar Scientific LTD, Stansted, UK). After fixation, cells were treated with 10 mM ethanolamine to block aldehydic groups and washed several times before plating OS cells. They were transfected with miR-CT3 mimic or NC, added to each well and incubated for 3 h at 37 °C-5% CO_2_. Non adherent cells were washed, and adhesion was detected by hematoxylin/eosin staining. The images are taken by using a Nikon Eclipse Ti microscope and adherent OS cells appeared round and dark, and are counted using the specific tools for measuring and counting provided by the software (Imaging Software NIS Elements BR). Each experimental condition was assayed in triplicate; the number of adherent OS cells was counted in 5 fields for each condition.

### 4.11. Soft-Agar Colony Formation Assay

OS cells transfected with miR-CT3 mimic or NC were assayed for their capacity to form colonies in soft-agar. Briefly, a total of 2 × 10^4^ cells were suspended in DMEM high glucose supplemented with 10% FBS containing 0.35% Noble agar (Sigma Chemical Co., St. Louis, MO, USA). Cells were plated on a layer of 0.7% Noble agar in DMEM (10% FBS) onto a 35-mm petri dish. Medium was refreshed every 5 days. On week 4, the number of colonies per field was counted under the contrast-phase Nikon Eclipse Ti microscope (Japan). Two independent experiments were carried out in triplicate.

### 4.12. ELISA Assay

Murine Matrix Metalloproteinase 9 (MMP9) and VEGFA levels secreted by OS cells were quantified by ELISA Kit for Quantikine Human MMP-9 ELISA Kit (DMP900) (R&D Systems, lnc., Minneapolis, MN, USA) and Quantikine Human VEGFA ELISA Kit (DVE00) (R&D Systems, lnc., Minneapolis, MN, USA). Briefly, supernatants from osteosarcoma cells transfected with miR-CT3 mimic or NC in DMEM high glucose supplemented with 10% FBS, were harvested after 48 h and analyzed.

### 4.13. Flow Cytometric Analysis

Percentage of apoptotic cells was assessed by Partec CyFlow Space Flow Cytometer (SYSMEX PARTEC GMBH, Goerlitz, Germany). Samples for apoptosis were collected by trypsin, washed twice with PBS, stained by 5 μL Propidium iodide (PI) and 5 μL FITC-Annexin V in 100 uL binding buffer for 15 min in dark. Fluorescent emissions were collected through FL1 band-pass filter for FITC-Annexin V, FL2 for PI. The apoptotic were PI and FITC-Annexin V both positive cells. SAOS-2 and MG-63 cells were stained with the following monoclonal antibodies: anti-human CD106 (VCAM-1)PE and anti-human CD326 (EP-CAM)BV605 (Pharmingen, BD Bioscience, Mountain View, CA, USA). All measurements were made with a FACS CELESTA flow cytometer (Becton Dickinson, San Jose, CA, USA) with the same instrument setting. At least 104 cells were analyzed using FACSDiva 8.0.1 (Becton Dickinson, San Jose, CA, USA) software.

### 4.14. Statistical Analysis

After having verified normal distribution of data, unpaired Student *t* test was used to compare experimental result to relative control one by using R software v.4.1.0 (R Core Team (2021). R: A language and environment for statistical computing. R Foundation for Statistical Computing, Vienna, Austria. URL https://www.R-project.org/, accessed on 9 December 2021).

## 5. Conclusions

The current study aimed to elucidate the role of the novel microRNA miR-CT3 in OS cells [28]; data obtained indicated miR-CT3 as a potential tumor suppressor miRNA, particularly affecting tumor angiogenesis and metastatic mechanisms.

**Figure 8:**
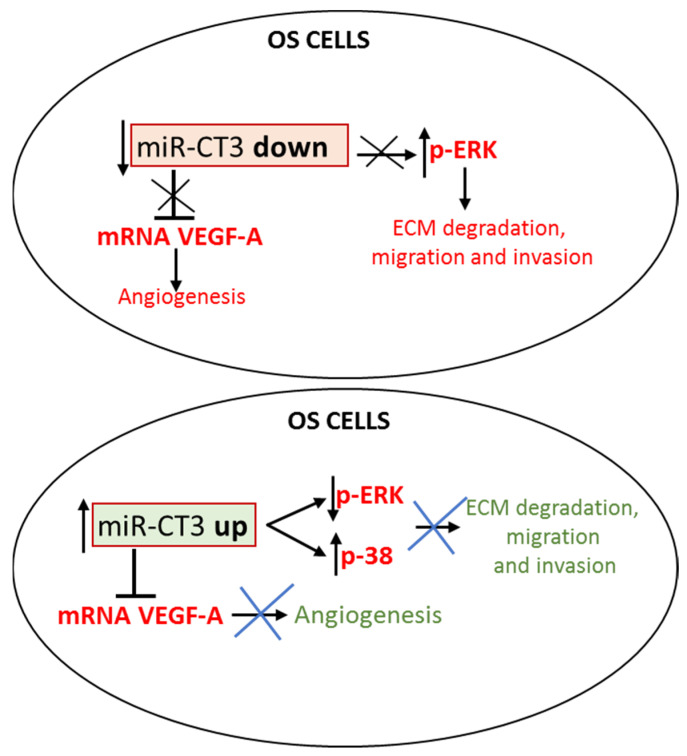
MiR-CT3 controls invasiveness in OS cells. Upper Figure: miR-CT3 expression is maintained low in OS cells thus allowing the expression of pro-angiogenic factors and the mechanisms of cellular invasion/migration. Lower Figure: when miR-CT3 expression is over-expressed, it can (1) binds and blocks the expression of mRNA VEGF-A, thus reducing tumor angiogenesis and (2) inhibits the phosphorylation of pERK1/2, activates the phosphorylation of p38 MAPK and controls the EMT-proteins expression, thus perturbating the mechanisms of cellular invasion/migration.

We hypothesize a possible molecular mechanism of miR-CT3, which might (1) control tumor angiogenesis by direct link with VEGF-A, passing also through the inactivation of the MAPK/ERK pathway, and (2) counteract the migration and invasion of OS cells, through the inhibition of some EMT proteins, such as Vimentin, passing through the activation of p38 MAPK pathway (Figure 8). Progressively, we will proceed to new in vitro studies on the other novel miRNAs identified through NGS analysis in OS cells [28] with the perspective of identifying a new miRNAs signature related to metastasis in OS.

## 6. Ethics Approval

For the collection of human blood samples two specific protocol studies were followed, which were approved by Bioethical Committee of the IFO_AOO—AOO—Istituti Fisioterapici Ospitalieri on 16 February 2017 (resolution number899/17) for osteosarcoma patients and by Ethical Committee AVEC (CE AVEC EM66-2020_352/2018/Sper/IOR_EM1 27/01/2020) for metastatic cancer patients. The study was conducted according to the guidelines of the Declaration of Helsinki. The aim of the study, study stages, and sample collection procedures were explained to all subjects. All subjects gave their written informed consent to participate in the study, including for blood sample collection and use of clinical data for research.

## Data Availability

The data presented in this study are available on request from the corresponding author.

## Supplementary Materials

The following are available online at https://www.mdpi.com/article/10.3390/ijms23020705/s1.
