# Supplementary material for: Potential Anti-Metastatic Role of the Novel miR-CT3 in Tumor Angiogenesis and Osteosarcoma Invasion
Source: Int J Mol Sci. 2022 Jan 9;23(2):705. doi: 10.3390/ijms23020705 (PMC8775549; doi:10.3390/ijms23020705)
Supplement: Supplementary file 1 [file ijms-23-00705-s001.zip › Figure S1.pdf]

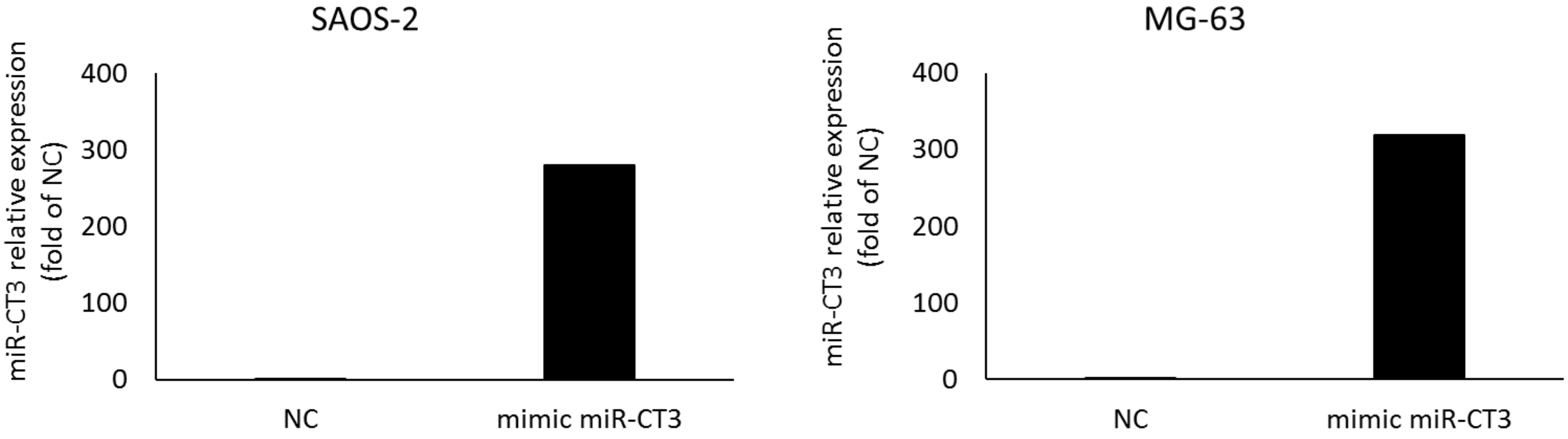

Figure S1.

miR-CT3 over-expression in OS cells (SAOS-2 and MG-63) transfected with miR-CT3 mimics negative control (NC). Values are expressed as fold of control (NC).
