# Supplementary material for: Potential Anti-Metastatic Role of the Novel miR-CT3 in Tumor Angiogenesis and Osteosarcoma Invasion
Source: Int J Mol Sci. 2022 Jan 9;23(2):705. doi: 10.3390/ijms23020705 (PMC8775549; doi:10.3390/ijms23020705)
Supplement: Supplementary file 1 [file ijms-23-00705-s001.zip › Figure S2.pdf]

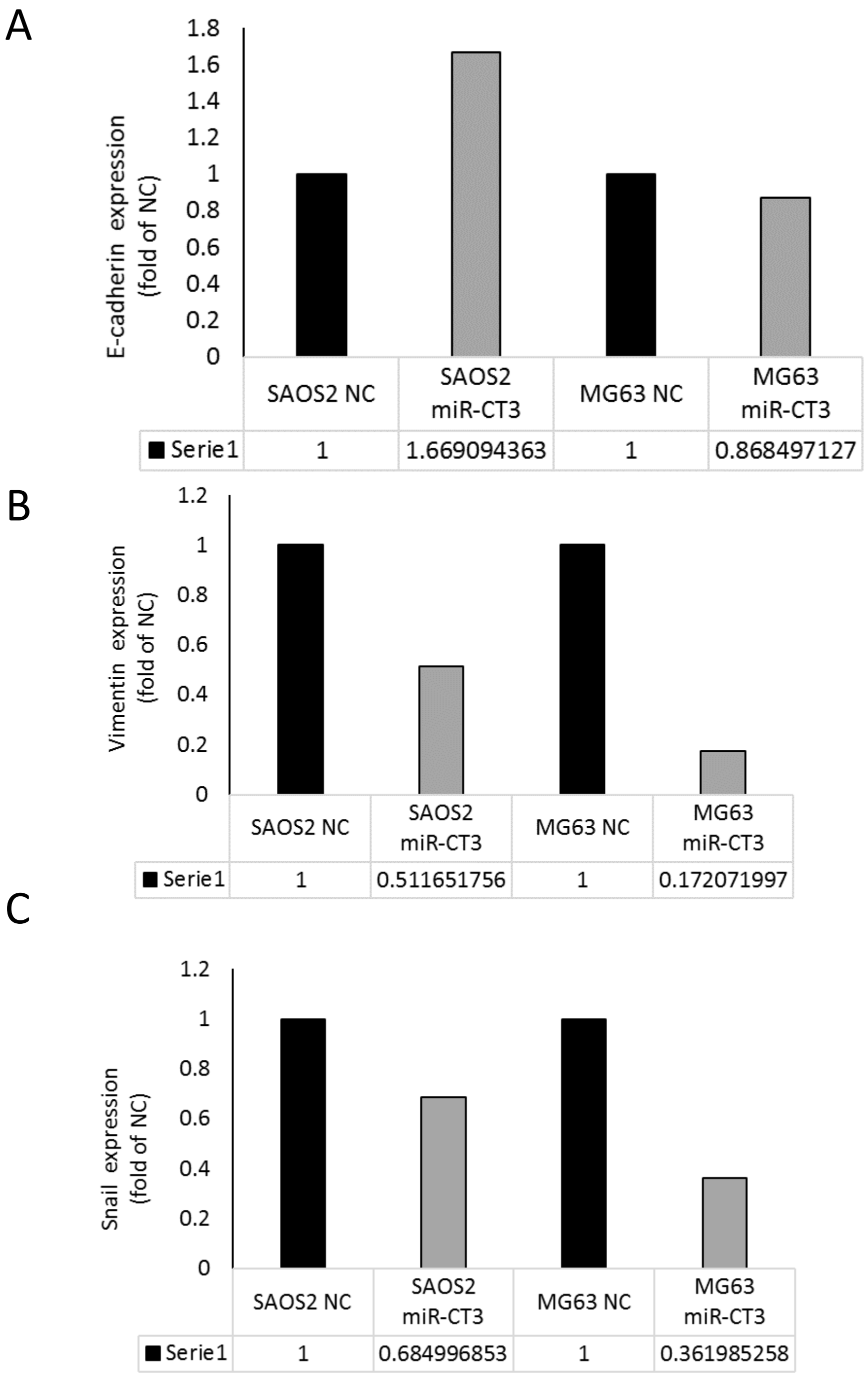

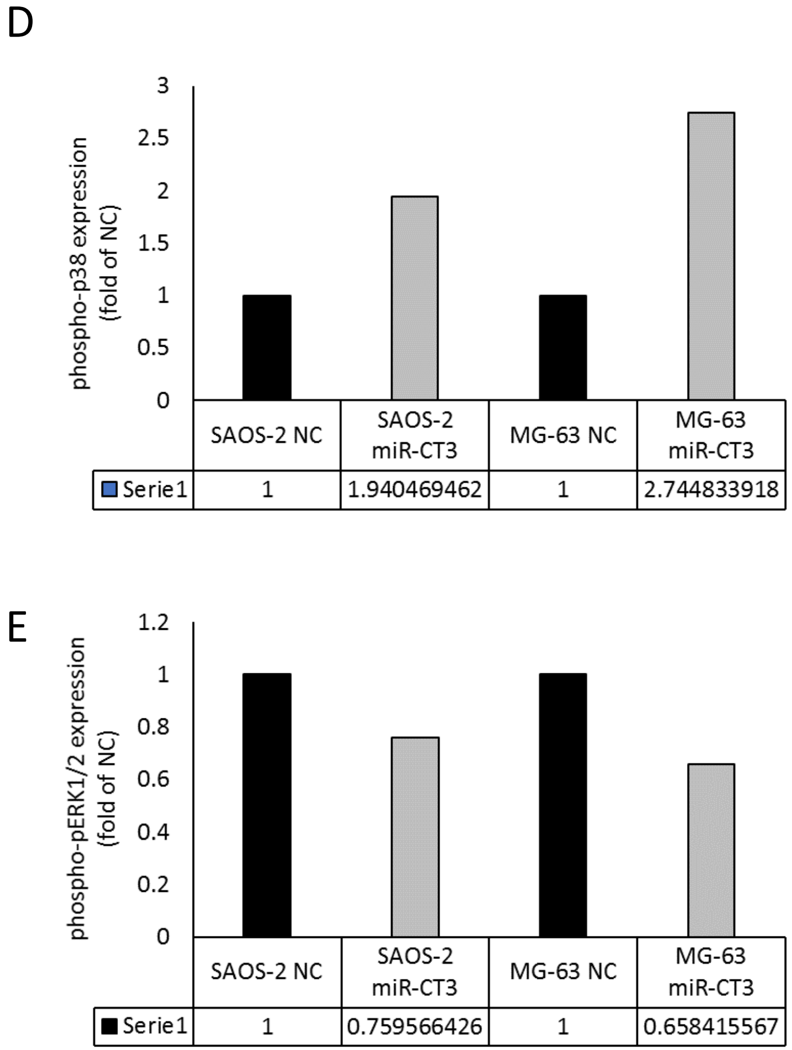

Figure S2

Densitometry analysis of western blotting of Figure 6A-B-C

A. Densitometry values (normalized to α-tubulin) of E-cadherin. B. Densitometry values (normalized to α -tubulin) of Vimentin. C. Densitometry values (normalized to α -tubulin) of Snail. D. Densitometry values (normalized to p38 total) of phospho-p38. E. Densitometry values (normalized to pERK1/2 total) of phospho-pERK1/2.

Figure 6A: WB

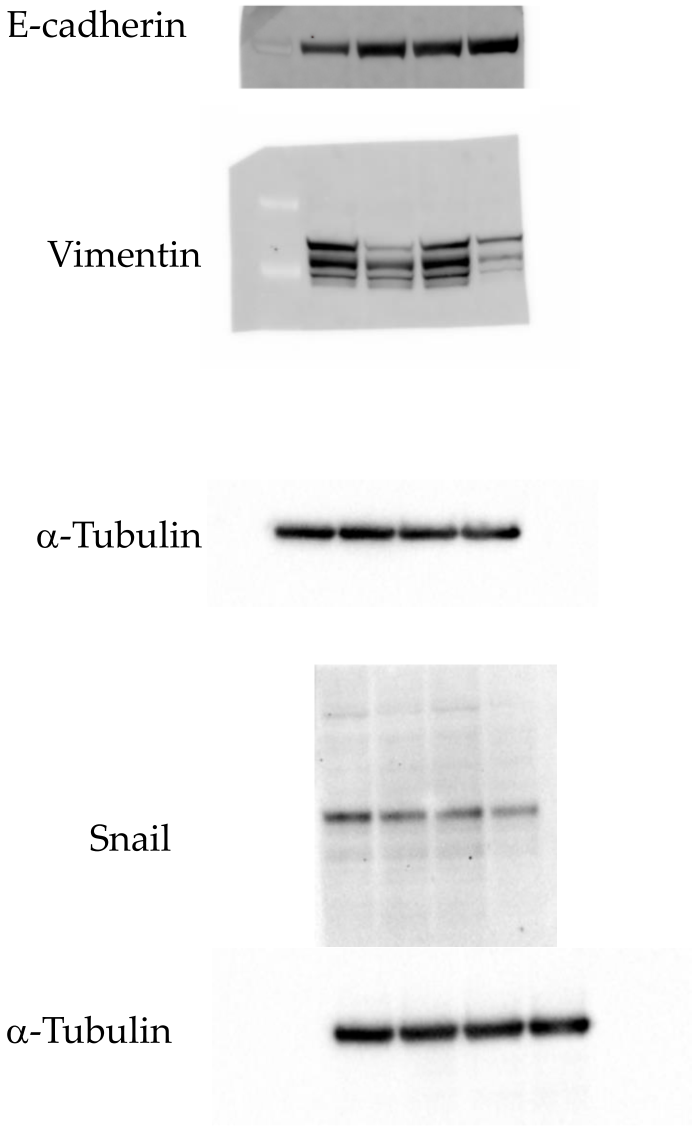

Figure 6B: WB phospho- and total-p38 and α-tubulin

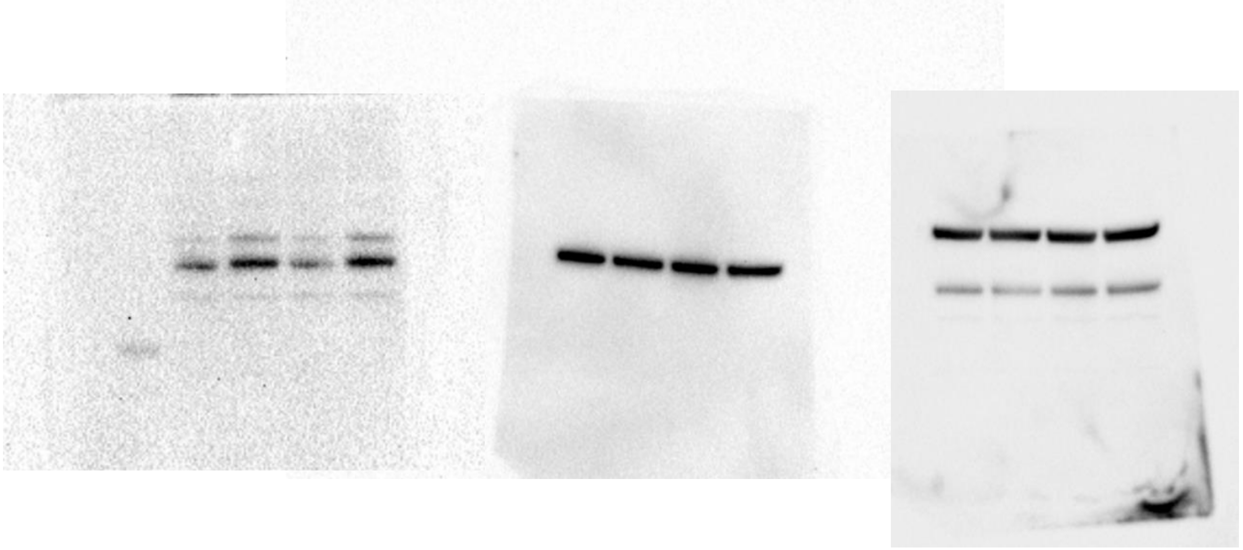

Figure 6C: WB phospho- and total-p42/44 ERK and GAPDH

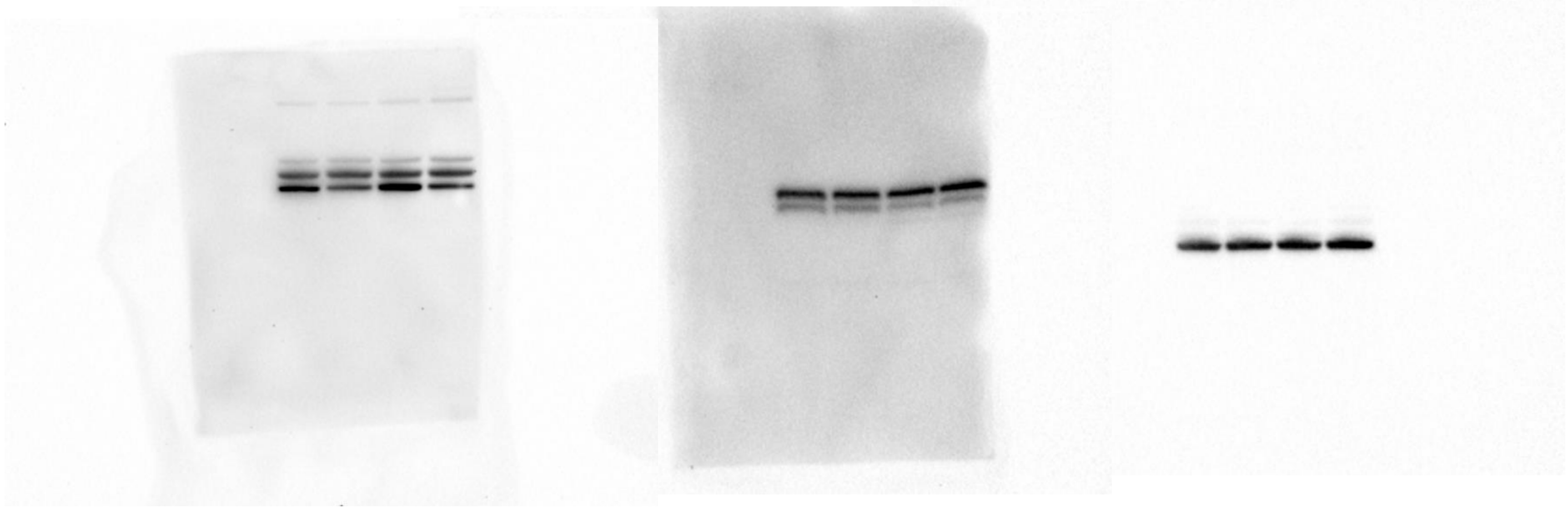

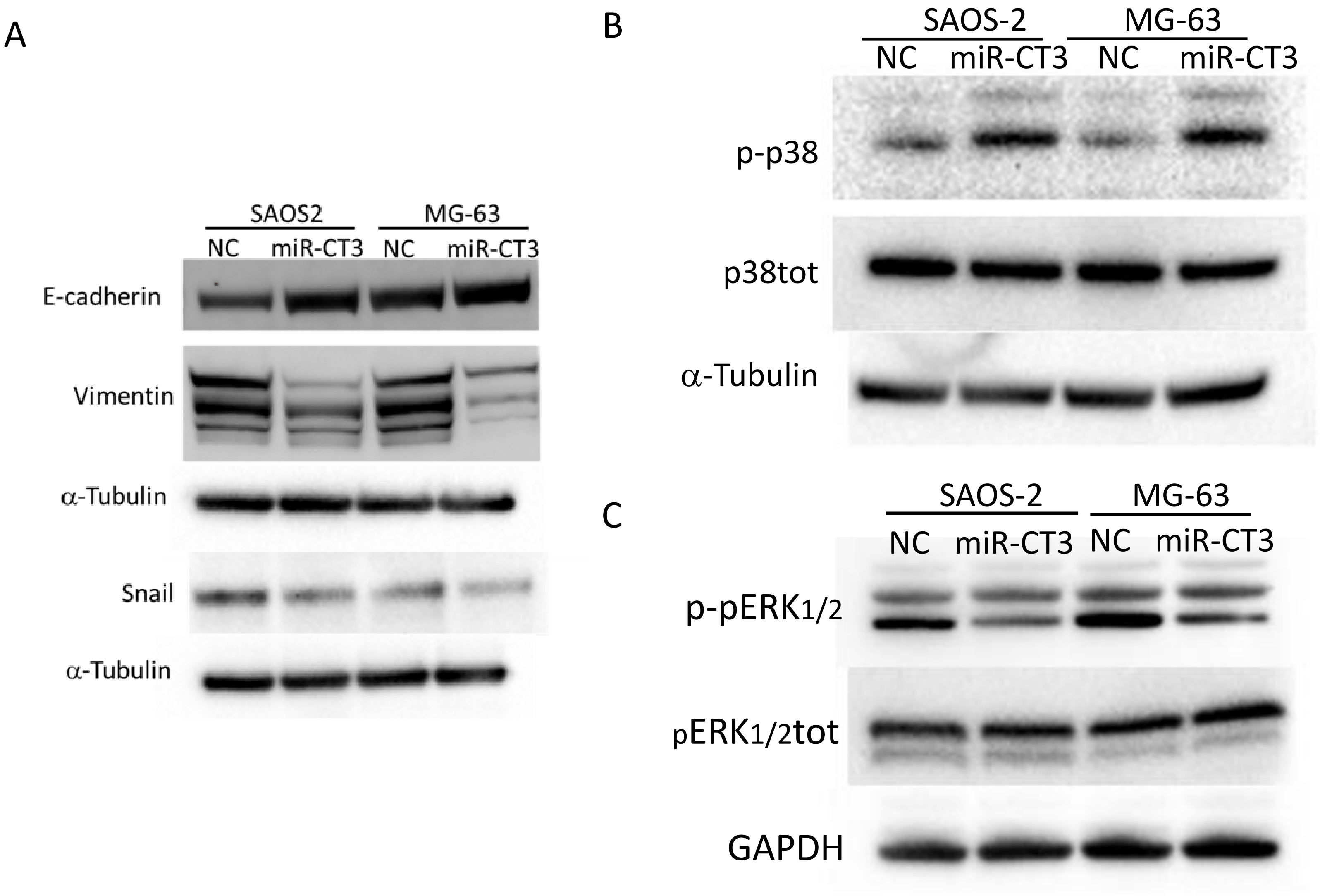

Figure 6. miR-CT3 over-expression reduces the expression of proteins of the EMT-pathway

A. Western blotting analysis of E-cadherin, Vimentin, Snail in SAOS-2 and MG-63 cells transfected with miR-CT3 mimics and negative control (NC) for 24 hours. Tubulin was used as loading control. B. Western blotting analysis of phospho- and total-p38 MAPK in SAOS-2 and MG-63 cells transfected with miR-CT3 mimics and negative control (NC) for 24 hours. Tubulin was used as loading control. C. Western blotting analysis of phospho- and total-p42/44 ERK in SAOS-2 and MG-63 cells transfected with miR-CT3 mimics and negative control (NC) for 24 hours. GAPDH was used as loading control.

Figure 6
