# Supplementary material for: Potential Anti-Metastatic Role of the Novel miR-CT3 in Tumor Angiogenesis and Osteosarcoma Invasion
Source: Int J Mol Sci. 2022 Jan 9;23(2):705. doi: 10.3390/ijms23020705 (PMC8775549; doi:10.3390/ijms23020705)
Supplement: Supplementary file 1 [file ijms-23-00705-s001.zip › Figure S3.pdf]

Figure S3

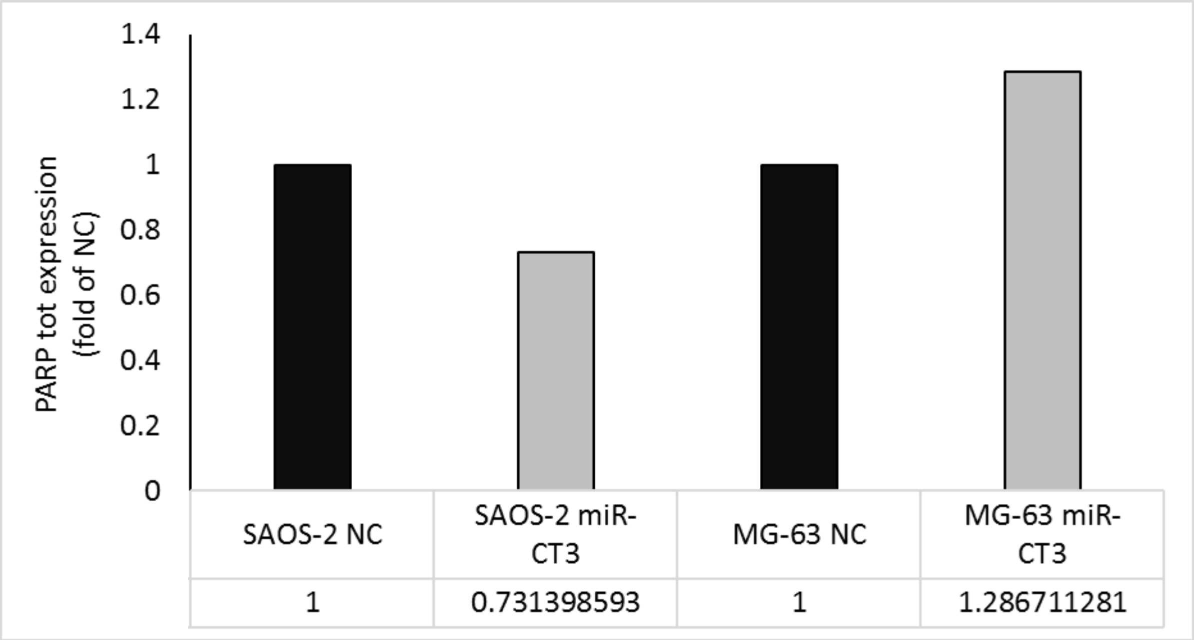

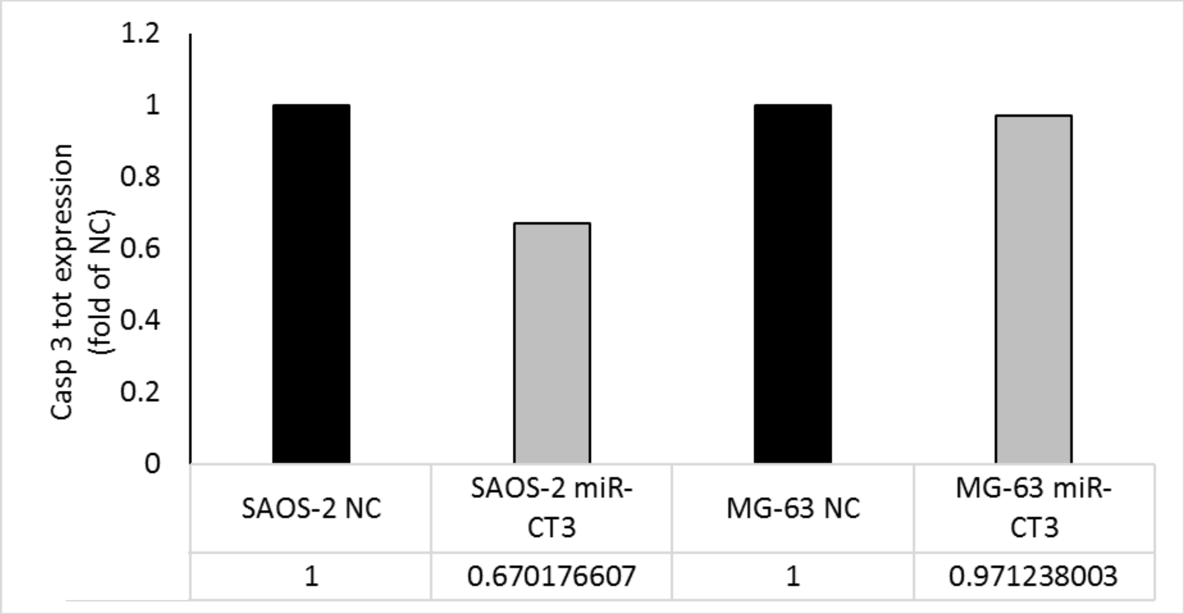

Densitometry analysis of western blotting of Figure 4C
Densitometry values (normalized to α-tubulin) of Parp tot and Casp3 tot.
