# Supplementary material for: Potential Anti-Metastatic Role of the Novel miR-CT3 in Tumor Angiogenesis and Osteosarcoma Invasion
Source: Int J Mol Sci. 2022 Jan 9;23(2):705. doi: 10.3390/ijms23020705 (PMC8775549; doi:10.3390/ijms23020705)
Supplement: Supplementary file 1 [file ijms-23-00705-s001.zip › Figure S4.pdf]

A

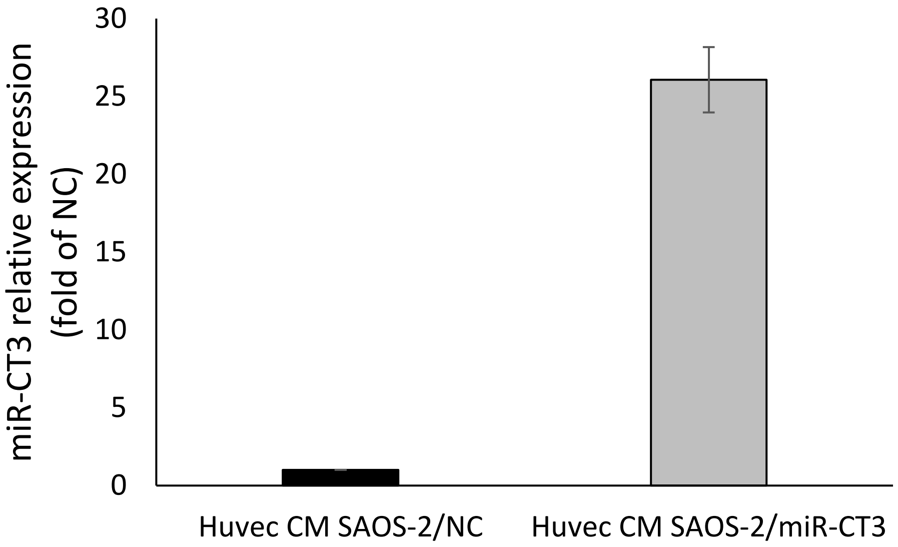

SF3 A. miR-CT3 expression in Huvec cells treated with the conditioned medium of SAOS-2 cells trasfected with miR-CT3 mimic or NC

B

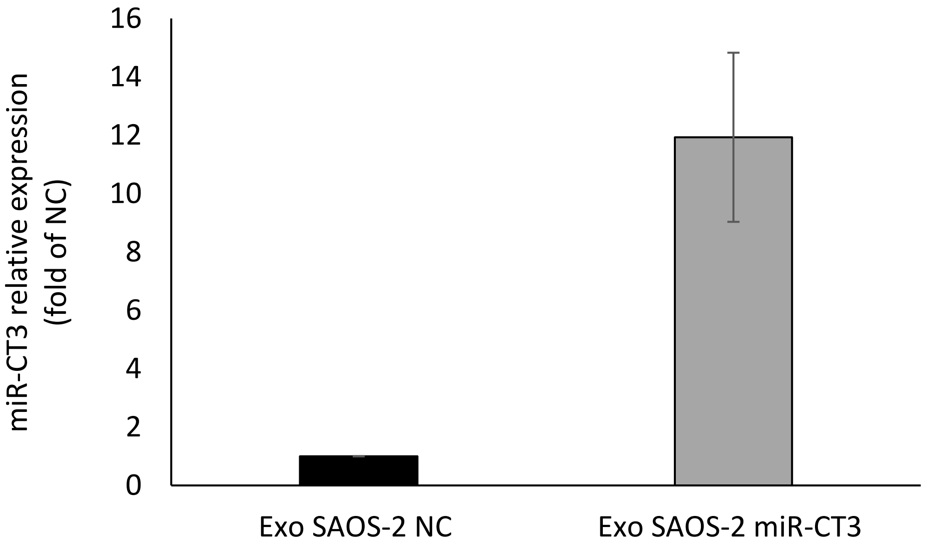

SF3 B. miR-CT3 expression in exosomes derived from SAOS-2 cells trasfected with miR-CT3 mimic or NC
