# Supplementary material for: Potential Anti-Metastatic Role of the Novel miR-CT3 in Tumor Angiogenesis and Osteosarcoma Invasion
Source: Int J Mol Sci. 2022 Jan 9;23(2):705. doi: 10.3390/ijms23020705 (PMC8775549; doi:10.3390/ijms23020705)
Supplement: Supplementary file 1 [file ijms-23-00705-s001.zip › Table S1.pdf]

Table S1. List of gene primers used to study gene expression profiling. Their expression was normalized to the β-actin

Gene	Forward primer sequence	Reverse primer sequence
human VEGF	CGAGGGCCTGGAGTGTGT	CGCATAATCTGCATGGTGATG
human MMP9	CGCTACCACCTCGAACTTTG	GCCATTCACGTCGTCCTTAT
human IL-8	GAATGGGTTTGCTAGAATGTGATA	CAGACTAGGGTTGCCAGATTTAAC
human β-actin	AGGCACCAGGGCGTGAT	GCCCACATAGGAATCCTTCTGAC
